# High value of mid‐regional proadrenomedullin in COVID‐19: A marker of widespread endothelial damage, disease severity, and mortality

**DOI:** 10.1002/jmv.26676

**Published:** 2021-02-19

**Authors:** Silvia Spoto, Felice E. Agrò, Federica Sambuco, Francesco Travaglino, Emanuele Valeriani, Marta Fogolari, Fabio Mangiacapra, Sebastiano Costantino, Massimo Ciccozzi, Silvia Angeletti

**Affiliations:** ^1^ Diagnostic and Therapeutic Medicine Department University Campus Bio‐Medico of Rome Rome Italy; ^2^ Intensive Care and Pain Management, Department of Anesthesia University Campus Bio‐Medico of Rome Rome Italy; ^3^ Emergency Department University Campus Bio‐Medico of Rome Rome Italy; ^4^ Unit of Clinical Laboratory Science University Campus Bio‐Medico of Rome Rome Italy; ^5^ Unit of Cardiovascular Science University Campus Bio‐Medico of Rome Rome Italy; ^6^ Unit of Medical Statistics and Molecular Epidemiology University Campus Bio‐Medico of Rome Rome Italy

**Keywords:** acute respiratory distress syndrome, adrenomedullin, COVID‐19, multiple organ dysfunction syndrome, SARS virus

## Abstract

The widespread endothelial damage due to severe acute respiratory syndrome coronavirus 2 (SARS‐CoV‐2) may lead to a disruption of the adrenomedullin (ADM) system responsible for vascular leakage, increased inflammatory status, and microvascular alteration with multi‐organs dysfunction. The aim of this study was to evaluate the role of mid‐regional proadrenomedullin (MR‐proADM) as a marker of SARS‐CoV2 related widespread endothelial damage, clinically identified by organs damage, disease severity and mortality. Patients with SARS‐CoV‐2 infection has been prospectively enrolled and demographic characteristic, clinical and laboratory data has been evaluated. In the overall population, 58% developed acute respiratory distress syndrome (ARDS), 23.3% of patients died, 6.5% acute cardiac injury, 1.4% of patients developed acute ischemic stroke, 21.2% acute kidney injury, 11.8% acute liver damage, and 5.4% septic shock. The best MR‐proADM cut‐off values for ARDS development and mortality prediction were 3.04 and 2 nmol/L, respectively. Patients presenting with MR‐proADM values ≥2 nmol/L showed a significantly higher mortality risk. In conclusion, MR‐proADM values ≥2 nmol/L identify those patients with high mortality risk related to a multiorgan dysfunction syndrome. These patients must be carefully evaluated and considered for an intensive therapeutic approach.

**Abbreviations**
ADM: adrenomedullin
ARDS: acute respiratory distress syndrome
AUC: areas under the curve
COVID‐19: coronavirus disease 2019
CRP: C‐reactive protein
MODS: multiple organ dysfunction syndrome
MR‐proADM: mid‐regional‐proadrenomedullin
PCT: procalcitonin
ROC: receiver operating characteristic
SARS‐CoV‐2: severe acute respiratory distress syndrome coronavirus 2
SOFA: sequential organ failure assessment

## 1 INTRODUCTION

The coronavirus disease 2019 (COVID‐19), caused by the severe acute respiratory distress syndrome coronavirus 2 (SARS‐CoV‐2), was responsible for an unprecedent threat to global health. ^1^ The clinical manifestations of the disease range from asymptomatic cases to severe pneumonia with high mortality rates (4%–13%), mainly in the case of acute respiratory distress syndrome (ARDS) development.^2^

Trying to stratify disease severity, the World Health Organization classified patients in four classes (mild to critical) basing on clinical and radiological characteristics.^3^ The use of biomarkers, however, may help clinicians identifying those patients with a severe disease and a higher risk of death.^4^, ^5^ Up to date, no markers of endothelial damage in COVID‐19 have been validated in clinical practice.

Adrenomedullin (ADM), a 6 kDa protein with a 22 min half‐life, is produced by endothelial and vascular smooth muscle cells due to volume overload to maintain endothelial barrier function, freely diffuses through the blood and interstitium, and binds to specific widespread receptors, mainly located in cardiovascular and pulmonary tissues.^6^, ^7^ The leading function of ADM is the vasodilatation in both vascular resistance and capacitance vessels resulting in a blood flow increase. ADM further reduce vasoconstriction through an inhibition of the renin‐angiotensin‐aldosterone system and maintains endothelial integrity reducing vascular permeability.^7^ A disruption of ADM system results in vascular leakage that represents the first step of inflammation and of coagulation cascade activation.^8^, ^9^

As derived from ADM in a 1:1 ratio, mid‐regional proadrenomedullin (MR‐proADM) values directly reflect the effects of its less stable and easily detectable precursor and has been recently introduced in clinical practice as a prognostic marker in patients with bacterial infection.^10^ A significant relation between MR‐proADM values and bacterial pneumonia severity index score, indeed, has been highlighted.^11^ Healthy individuals showed MR‐proADM values of about 0.33 nmol/L.^10^ MR‐proADM values of 0.8 nmol/L, conversely, are diagnostic of bacterial infection with higher values indicative of a higher infection severity from 1.2 to 1.9 and 3.7 nmol/L in localized infections, sepsis or septic shock, respectively‐.^12^ In patients with sepsis and septic shock, MR‐proADM values more than 3.4 and 4.3 nmol/L, respectively, were significantly associated with 90‐day mortality.^13^

Despite the vast majority of studies evaluated the role of MR‐proADM in bacterial infections leading to sepsis, scant evidence is available in patients with viral infections without any information on COVID‐19.^12^, ^14^, ^15^, ^16^

Knowing that COVID‐19 related damage resemble the alteration occurring during sepsis, however, a disruption of ADM pathway during SARS‐CoV‐2 infection may be hypothesized.^8^, ^9^, ^17^

The aim of this study was to evaluate the role of MR‐proADM as a marker of SARS‐CoV‐2 related widespread endothelial damage, clinically identified by organs damage, disease sevrity, and mortality.

## 2 MATERIALS AND METHODS

This study has been approved by the Ethical Committee of the University Campus Bio‐Medico of Rome and all patients provided informed consent before the enrollment within the study.

### 2.1 Patient selection and characteristics

All patients hospitalized for SARS‐CoV‐2 infection at COVID Center of the Campus Bio‐Medico of Rome University, were prospectively included between 1st April and 30th June 2020. The COVID center included both medicine department and intensive care unit (ICU). Pregnancy and lack of informed consent represented exclusion criteria.

The following data were collected at inclusion: demographic characteristics (age and gender); onset symptoms; relevant comorbidities; immune status (active malignancy or other causes of immunosuppression); concomitant antimicrobial, antiviral, or immunosuppressive treatments administration; clinical presentation. Furthermore, all patients received a complete physical examination including body temperature, blood pressure, heart and respiratory rate, cardiac, pulmonary, abdominal, and neurological evaluation. Laboratory values at inclusion comprehended complete blood counts, MR‐proADM, C‐reactive protein (CRP), ferritin, procalcitonin (PCT), coagulation ( *d* ‐dimer, international normalized ratio, activated partial thromboplastin time), liver (aspartate aminotransferase, alanine aminotransferase, albumin, bilirubin) and kidney (creatinine) functionality tests, serum lactate, arterial blood gas examination.

All patients received standard of care basing on disease severity and comprehending oxygen support, anticoagulant therapy, hydroxychloroquine, and tocilizumab whether indicated.

### 2.2 Laboratory values measurement

Diagnosis of COVID‐19 was confirmed by a reverse transcription polymerase chain reaction test on a nasopharyngeal and/or endotracheal aspirate swab detecting spike protein (S) and envelope (E) genes for SARS‐CoV‐2. MR‐proADM and PCT plasma concentrations were measured by an automated Kryptor analyzer, using a time‐resolved amplified cryptate emission technology assay (Kryptor PCT; Brahms AG), with commercially available immunoluminometric assays (Brahms).^12^, ^18^, ^19^, ^20^

### 2.3 Clinical outcomes and definitions

Primary outcome was ARDS development and 30‐day mortality. Secondary outcomes were acute cardiac injury, transient ischemic attack or stroke, acute kidney injury, acute liver failure, and septic shock development. ARDS was defined according to the Berlin definition; acute cardiac injury when there was a rise and/or fall of cardiac troponin values with at least one value above the 99th percentile of the upper reference limit; transient ischemic stroke as a brief episodes of neurological dysfunction resulting from focal cerebral ischemia not associated with permanent cerebral infarction; acute ischemic stroke as an episode of neurological dysfunction caused by focal cerebral, spinal, or retinal infarction; acute kidney injury diagnosed by KDIGO criteria as an increase in serum creatinine by ≥0.3 mg/dl within 48 h, ≥ 1.5 times from baseline within 7 days, or urine volume <0.5 ml/kg/h for 6 h; acute liver damage as elevation of serum transaminases (>2 x upper normal values); shock as persisting hypotension despite volume resuscitation, requiring vasopressors to maintain mean arterial pressure ≥65 mmHg and serum lactate level >2 mmol/L.^21^, ^22^, ^23^, ^24^, ^25^, ^26^

All included patients were followed until death or 30‐day follow‐up, whichever came first.

### 2.4 Statistical analysis

Continuous variables were expressed as mean (standard deviation) or median (interquartile ranges), according to data distribution, and were compared using the Student's *t* test or the Mann–Whitney *U* test; categorical variables were expressed as counts and percentages and compared using the *χ* ^2^ or Fisher's exact tests, as appropriate.

Receiver operating characteristic (ROC) analysis has been performed among independent variables associated with SARS‐CoV‐2 infection to define the cutoff point for MR‐proADM, CRP, ferritin, PCT values, and sequential organ failure assessment (SOFA) score in predicting ARDS and mortality, and the accuracy of MR‐proADM, CRP, Ferritin, and PCT values in patients with SARS‐CoV‐2 infection.

ROC curves and areas under the curve (AUC) values has been calculated for all markers including a group of 50 healthy individuals evaluated at Campus Bio‐Medico University of Rome.

Pretest odds, posttest odds, and the consequent posttest probability have been computed to investigate whether combination of MR‐proADM, PCT, and SOFA score improves posttest probability.

Kaplan–Meier curves were created to estimate the overall survival and compared using the log‐rank test. To evaluate whether the value of MR‐proADM influenced mortality rates, a Cox regression model was fitted using age and sex as covariates and the adjusted hazard ratios were calculated.

Data have been analyzed using Med‐Calc 11.6.1.0 statistical package (MedCalc Software) and R (version 3.6.3, R Core Development Team).^27^

*p* < .05 were considered statistically significant.

## 3 RESULTS

### 3.1 Patients characteristics

A total of 69 patients has been included in the primary analysis. The main patient characteristics are shown in Table 1. The median age was 78.0 years and 53,6% of patients were male. Cardiovascular (68.1%) and chronic pulmonary disease (33.3%) represented the most frequent comorbidities. The median SOFA score was 2 (interquartile range [IQR], 1–7), 56.5% of patients has been admitted to medical ward while the 43.5% to ICU. Median hospital stay was 17 days.

**Table 1:** Characteristics of the study population

**Variables**	**Overall *n* = 69**	**MR‐proADM < 2, *n* = 43**	**MR‐proADM ≥ 2, *n* = 21**	***p* Value**
Median age, years (IQR)	78.00 (61.00–84.00)	72.00 (57.50–83.00)	81.00 (78.00–86.00)	.085
Male sex, *n* (%)	37 (53.6)	22 (51.2)	12 (57.1)	.854
Comorbidities, *n* (%)				
Cardiovascular	47 (68,1)	24 (60.0)	18 (94.7)	.014
Chronic pulmonary disease	23 (33.3)	11 (27.5)	8 (42.1)	.410
Chronic liver disease	4 (5.8)	3 (7.5)	0	.544
Kidney disease	13 (18.8)	3 (7.5)	9 (50.0)	.001
Diabetes mellitus	19 (27.5)	10 (25.0)	7 (38.9)	.445
Blood hypertension	38 (55.1)	21 (52.5)	13 (72.2)	.262
Active cancer	7 (10.1)	3 (7.5)	2 (11.8)	.629
Symptoms at onset, *n* (%)				
Fever	27 (39.1)	17 (48.6)	8 (42.1)	.866
Cough	15 (21.7)	13 (35.1)	2 (10.5)	.099
Dyspnea	26 (37.7)	10 (27.0)	13 (68.4)	.007
Pharyngodynia	3 (4.3)	3 (8.1)	0	.516
Gastrointestinal	11 (15.9)	6 (16.2)	3 (15.8)	1.000
Neurological	4 (5.8)	1 (2.7)	2 (10.5)	.263
Arthro‐myalgia	5 (7.2)	5 (13.5)	0	.155
Anosmia	69 (100.0)	37 (100.0)	19 (100.0)	NA
Laboratory values, median (IQR)				
MR‐proADM	1.49 (0.67–2.26)	0.91 (0.51–1.49)	4.19 (2.28–5.95)	<.001
CRP	4.24 (1.06–10.13)	2.71 (0.51–6.24)	6.80 (4.99–14.07)	.001
Ferritin	413.0 (125.5–1016.5)	245.5 (119.0–457.5)	777.5 (449.8–2009.0)	<.001
PCT	0.06 (0.03–0.41)	0.04 (0.03–0.06)	0.72 (0.09–6.83)	<.001
AST	27.50 (20.00–46.25)	25.00 (20.00–33.25)	46.00 (31.00–78.00)	.002
ALT	17.00 (9.75–31.00)	16.50 (9.25–29.75)	17.00 (10.00–49.00)	.682
Bilirubin	0.50 (0.40–0.80)	0.50 (0.40‐0.70)	0.60 (0.40–0.90)	.422
Creatinine	0.94 (0.70–1.50)	0.86 (0.66–1.01)	1.08 (0.99–3.68)	<.001
PaO_2_/FiO_2_	332.50 (237.00–383.25)	347.50 (281.75–390.00)	267.00 (197.00–381.00)	.147
Prognostic score and outcomes				
ICU admission, *n* (%)	30 (43.5)	13 (30.2)	13 (61.9)	.031
Hospital discharge, *n* (%)	53 (76.8)	41 (95.3)	9 (42.9)	<.001
Median hospital stays, days (IQR)	17.00 (9.00–32.00)	16.00 (10.00–25.00)	23.50 (13.25–44.25)	.054
SOFA (median [IQR])	2.00 (1.00–7.00)	1.00 (1.00–3.00)	7.00 (3.00–8.00)	<.001
ARDS, *n* (%)	40 (58.0)	22 (51.2)	15 (71.4)	.203
Acute cardiac injury, *n* (%)^a^	3 (6.5)	0	3 (14.3)	.108
Stroke	1 (1.4)	0	1 (1.4)	.108
Acute kidney injury, *n* (%)^a^	14 (21.2)	1 (3.3)	10 (32.3)	.009
Acute liver damage, *n* (%)^a^	8 (11.8)	2 (6.2)	6 (19.4)	.237
Septic shock, *n* (%)^a^	3 (5.4)	0	2 (13.3)	.082
Death, *n* (%)	16 (23.2)	2 (4.7)	12 (57.1)	<.001

Abbreviations: ARDS, acute respiratory distress syndrome; CRP, C‐reactive protein; ICU, intensive care unit; IQR, interquartile range; MR‐proADM, mid‐regional proadrenomedullin; PCT, procalcitonin; SOFA, sequential organ failure assessment.

^a^ These outcomes are not available for all included patients (please refer to the text).

In the overall population, 58% (40/69 patients) developed ARDS, 23.2% (16/69) of patients died, 6.5% (3/46) acute cardiac injury, 1.4% (1/69) of patients developed acute ischemic stroke, 21.2% (14/66) acute kidney injury, 11.8% (8/68) acute liver damage, and 5.4% (3/56) septic shock. At the end of follow‐up, all remaining 53 patients have been discharged.

### 3.2 Laboratory markers values in SARS‐CoV‐2 infection

Median MR‐proADM, CRP, ferritin, and PCT values were 1.49 nmol/L (IQR, 0.67–2.26), 4.24 mg/dl (IQR, 1.06–10.13), 413.00 ng/ml (IQR 125.5–1016.5), and 0.06 ng/ml (IQR, 0.03–0.41), respectively (Table S1).

AUCs values resulting from ROC curve analysis for MRproADM, CRP, PCT, and ferritin in patients with SARS‐CoV‐2 infection are showed in Table S2. ROC curves and AUC values resulted statistically significant for all variable, but PCT (Figure S1, Table S2).

The best cut‐off values for MR‐proADM, CRP, ferritin, and PCT in patients with SARS‐CoV‐2 infection were 1.00 nmol/L, 0.48 mg/dL, 115.58 ng/mL, and 0.26 ng/ml, respectively (Table S2).

ROC curves comparison between the different variables has been reported in Table S3 and schematized in Figure S1. AUC value for MR‐proADM (0.78) was significantly higher than PCT (0.55; *p* < .0001), smaller than CRP (0.91, *p* < .0001) and similar than ferritin (0.86, *p* = .051). AUC value of CRP was significantly higher than MR‐proADM and PCT (*p* < .0001) and similar than ferritin (*p* = .67). Finally, AUC value of ferritin was significantly higher only than PCT (*p* < .0001).

### 3.3 ARDS prediction during SARS‐CoV‐2 infection

Median values with interquartile ranges and Mann–Whitney's comparison for MR‐proADM, CRP, ferritin and SOFA score for patients with or without ARDS development during follow‐up are reported in Table S4. All these variables resulted significantly higher in patients with ARDS.

**Figure 1:**
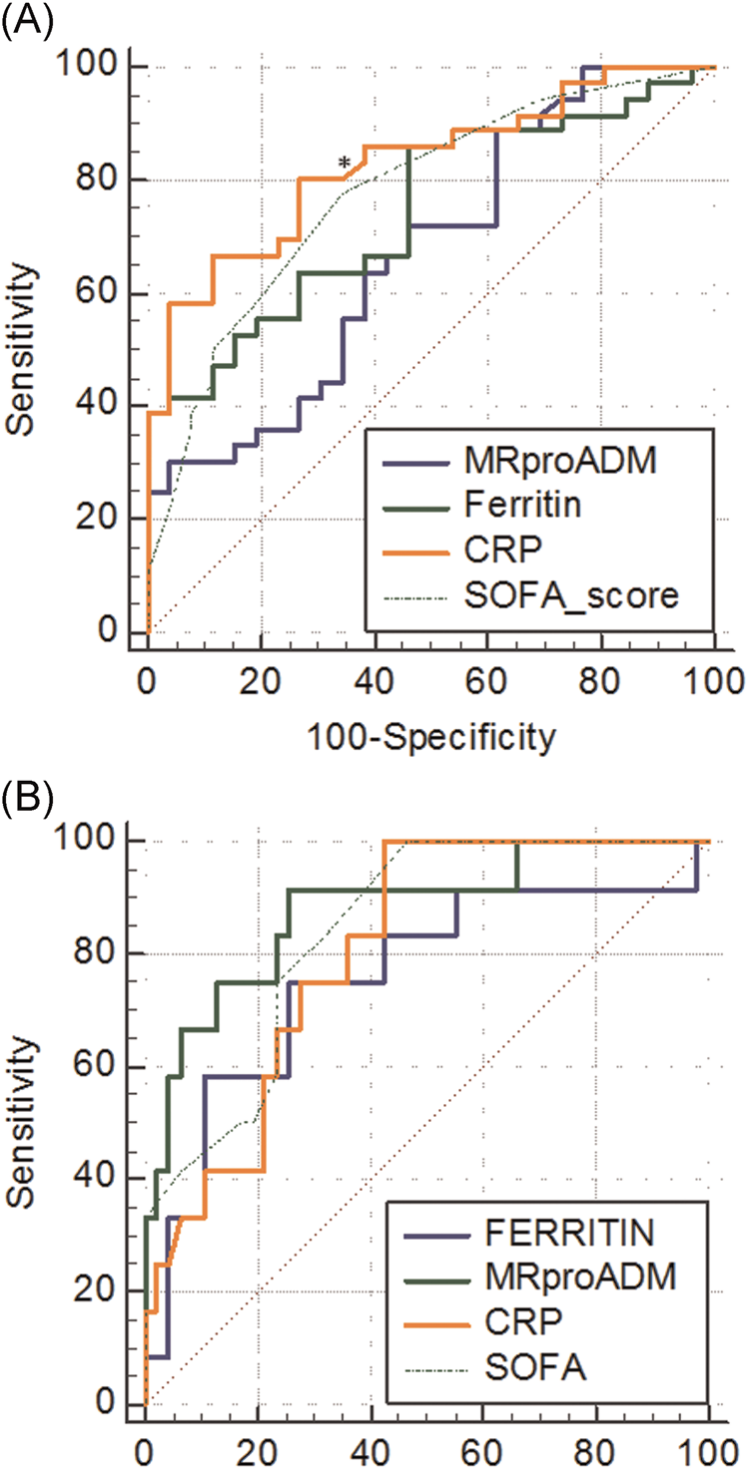
ROC curves for (A) ARDS development and (B) mortality in SARS‐CoV‐2 infection. ARDS, acute respiratory distress syndrome; ROC, receiver operating characteristic; SARS‐CoV‐2, severe acute respiratory syndrome‐coronavirus 2; SOFA, sequential organ failure assessment

ROC curves and AUC values resulted statistically significant for all considered variables despite only CRP presented significantly higher AUC values than MR‐proADM (*p* = .030) (Figure 1A, Figure S2, and Table S5 and S6). Furthermore, the best cut‐off for ARDS development prediction were 3.04 nmol/L for MR‐proADM, 3.88 mg/dl for CRP, 165.58 ng/ml for ferritin, and 1 for SOFA, respectively.

### 3.4 30‐Day mortality prediction during SARS‐CoV‐2 infection

Median values with interquartile ranges and Mann–Whitney's comparison for MR‐proADM and SOFA score in survivors and non survivors at 30‐day follow‐up are reported in Table S7.

ROC curve and AUC values for MR‐proADM and SOFA score resulted statistically significant (*p* < .0001) without differences between the variables (Figure 1B, Figure S3, and Tables S8 and S9). The best cut‐off for 30‐day mortality prediction were ≥2 nmol/L for MR‐proADM, 2.91 mg/dl for CRP, 635.86 ng/ml for ferritin, and ≥3 for SOFA score.

**Figure 2:**
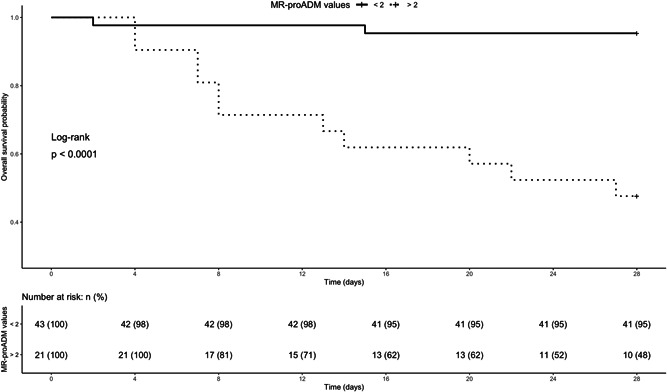
Kaplan–Meier curves in patients with MR‐proADM values < or ≥ 2 nmol/L. MR‐proADM, mid‐regional proadrenomedullin

Patients presenting with MR‐proADM values ≥2 nmol/L, indeed, showed a significantly higher mortality risk than patients with MR‐proADM values <2 nmol/L (adjusted hazard ratio 12.34; 95% confidence interval, 2.66–57.28; Figure 2 and Table S10).

## 4 DISCUSSION

The results of this study showed that MR‐proADM may be used as a marker of organ damage, disease severity, and mortality in patients with COVID‐19. Patients who developed ARDS, the most frequent complication, presented higher MR‐proADM values than patients without acute respiratory involvement. Furthermore, MR‐proADM values ≥2 nmol/L were associated with a significantly higher mortality risk.

COVID‐19 represents a systemic disease causing widespread endothelial damage with multiple organ dysfunction syndrome, in severe cases.^2^ The role of endothelial cells in organ failure development during infections has been recently evaluated.^9^ Coating the blood vessels and representing the interface between blood and parenchymal cells, vascular endothelial cell lining is responsible for organ function. The effects of endothelial cell lining are also supported by the glycocalyx that controls hemostasis, leukocyte, and platelet adhesion, the transmission of shear stress to the endothelium, and anti‐inflammatory defenses. A disruption of this system may occur during sepsis and result in organ dysfunction, mainly affecting the hemostatic, pulmonary, kidney, and liver systems.^9^, ^28^ Whether these alterations in endothelial cell lining is adaptive or maladaptive depends on both disease extension and time from disease onset. A localized vasodilatation, indeed, allows leukocytes to reach the site of infection while the activation of coagulation helps in restrain the widespread of infection. At more advanced stages, these alterations lead to a septic phenotype, resulting in a systemic reduction of vascular tone, increase in vascular permeability, alterations in microvascular perfusion, and hemostatic alteration up to disseminated intravascular coagulopathy.^9^ Furthermore, vascular endothelial damage along with blood hypercoagulability are well known risk factors for venous thromboembolism.^29^, ^30^

**Figure 3:**
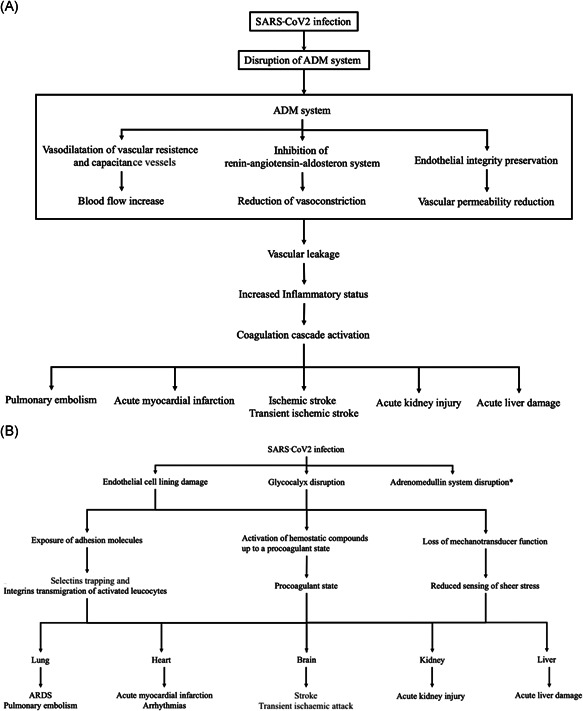
(A) Adrenomedullin system disruption and (B) widespread endothelial damage in SARS‐CoV‐2 infection. ADM, adrenomedullin; ARDS, acute respiratory distress syndrome; SARS‐CoV‐2, severe acute respiratory syndrome‐coronavirus 2. *see panel A

Being responsible for endothelial integrity, an alteration of ADM system during sepsis causes vascular leakage and organ dysfunction (Figure 3).^31^ Recent observational studies confirmed these pathological data showing as high values of ADM and of its more stable product MR‐proADM are significantly associated with organ failure, disease severity and a worse prognosis.^12^, ^16^, ^32^, ^33^

The widespread endothelial and pulmonary damage related to SARS‐CoV‐2 infection may cause a relevant disruption of the ADM system, mainly in severe cases. The receptors and binding sites for ADM, indeed, were mostly represented within the cardiovascular and lung tissue.^7^ Our results confirm these hypothesis and showed as MR‐proADM, identifying those patients with a higher risk of ARDS development and with a widespread organ involvement, may be listed among other evaluated prognostic markers.^34^

Furthermore, the role of ADM in COVID‐19 related organ damage may suggest the use of new therapeutic agents, such as monoclonal antibody. Adrecizumab, a humanized, monoclonal, non‐neutralizing ADM‐binding antibody has been evaluated in patients with sepsis and acute heart failure to improve vascular integrity, tissue congestion, and thereby clinical outcomes.^7^, ^35^

## 5 CONCLUSION

MR‐proADM values ≥2 nmol/L identify those patients with high mortality risk related to a multiple organ dysfunction syndrome. These patients must be carefully evaluated and considered for an intensive therapeutic approach. Further studies in larger populations will be warranted to confirm these data.

## CONFLICT OF INTERESTS

The authors declare that there are no conflict of interests.

## AUTHOR CONTRIBUTIONS

Silvia Spoto led the study design, data collection, data analysis, data interpretation, and manuscript writing. Felice E. Agrò, Federica Sambuco, and Francesco Travaglino assisted with data collection and analysis of the validation dataset. Emanuele Valeriani and Marta Fogolari assisted with computer queries, data analysis and manuscript preparation. F.M. assisted with data collection and analysis of the validation dataset. Massimo Ciccozzi assisted with data collection and analysis of the development dataset as well as study design, data interpretation and manuscript writing. Sebastiano Costantino and Silvia Angeletti assisted with chart review and data analysis and supervised all aspects of the investigation, as well as assisting with study design, data interpretation, and manuscript writing.

## Supporting information

Supporting information.

## Data Availability

The data that support the findings of this study are available from the corresponding author upon reasonable request.
