# Supplementary material for: High value of mid‐regional proadrenomedullin in COVID‐19: A marker of widespread endothelial damage, disease severity, and mortality
Source: J Med Virol. 2021 Feb 19;93(5):2820–7. doi: 10.1002/jmv.26676 (PMC7753433; doi:10.1002/jmv.26676)
Supplement: Supplementary file 1 — Supporting information. [file JMV-93-2820-s001.docx]

**Supplementary Table 1.** Median laboratory values in patients with SARS-CoV2 infection and control group.

| Variables | SARS-CoV2 | Control group | p-value^*^ |
| --- | --- | --- | --- |
| MR-proADM, nmol/L (IQR) | 1.49 (0.67-2.26) | 0.54 (0.42-0.76) | p<0.0001 |
| CRP, mg/dL (IQR) | 3.48 (0.70-8.33) | 0.15 (0.09-1.28) | p<0.0001 |
| Ferritin, ng/mL (IQR) | 413.70 (123.87-1023.25) | 59.53 (31.80-80.29) | p<0.0001 |
| PCT, ng/mL (IQR) | 0.06 (0.03-0.48) | 0.05 (0.045-0.075) | p=0.33 |

**^*^**Mann-Whitney Test

**Supplementary Table 2.** Accuracy of cut-off values for laboratory markers in patients with SAR-CoV2 infection.

| Variables | Cut-off | Sensitivity | Specificity | +LR | -LR | AUC | p-value |
| --- | --- | --- | --- | --- | --- | --- | --- |
| MR-proADM, nmol/L | >1 | 64,06 | 88,00 | 5,34 | 0,41 | 0.78 | <0.0001 |
| CRP, mg/dL | >0,48 | 82,09 | 95,92 | 20,11 | 0,19 | 0.91 | <0.0001 |
| Ferritin, ng/mL | >115 | 79,37 | 89,80 | 7,78 | 0,23 | 0.86 | <0.0001 |
| PCT, ng/mL | >0,26 | 29,85 | 94,00 | 4,98 | 0,75 | 0.55 | 0.33 |

**Supplementary Table 3.** Pairwise comparison of ROC curves for laboratory markers in patients with SAR-CoV2 infection

| MRproADM ~ PCT | |
| --- | --- |
| Difference between areas | 0,243 |
| Significance level | p < 0,0001 |
| MRproADM ~ CRP | |
| Difference between areas | 0,123 |
| Significance level | p = 0,0101 |
| MRproADM ~ FERRITIN | |
| Difference between areas | 0,105 |
| Significance level | p = 0,0512 |
| PCT ~ CRP | |
| Difference between areas | 0,366 |
| Significance level | p < 0,0001 |
| PCT ~ FERRITIN | |
| Difference between areas | 0,348 |
| Significance level | p < 0,0001 |
| CRP ~ FERRITIN | |
| Difference between areas | 0,0180 |
| Significance level | p = 0,6753 |

**Supplementary Table 4.** Median laboratory values in patients in patients with or without ARDS development during follow-up

| Variables | With ARDS | Without ARDS | p-value^*^ |
| --- | --- | --- | --- |
| MR-proADM, nmol/L (IQR) | 1.75 (0.94-4.77) | 1.05 (0.50-1.87) | p=0.0053 |
| CRP, mg/dL (IQR) | 6.58 (2.97-13.10) | 0.95 (0.30-3.33) | p<0.0001 |
| Ferritin, ng/mL (IQR) | 624.87 (245.20- 1774.10) | 151.00 (66.53-482.86) | p=0.0006 |
| SOFA score (IQR) | 4 (2-7) | 1 (0-2) | p=0.0001 |

**Supplementary Table 5.** Accuracy of cut-off values of laboratory markers for ARDS development

| Variables | Cut-off | Sensitivity | Specificity | +LR | -LR | AUC | p-value |
| --- | --- | --- | --- | --- | --- | --- | --- |
| MR-proADM, nmol/L | >3 | 37.50 | 86.21 | 2.72 | 0.73 | 0.70 | 0.002 |
| CRP, mg/dL | 3.88 | 69.23 | 85.71 | 4.85 | 0.36 | 0.83 | <0.0001 |
| Ferritin, ng/mL | >165.60 | 86.11 | 55.56 | 1.94 | 0.25 | 0.75 | <0.0001 |
| SOFA score | >1 | 80.00 | 62.96 | 2.16 | 0.32 | 0.78 | <0.0001 |

| MR-proADM ~ SOFA score | |
| --- | --- |
| Difference between areas | 0.10 |
| Significance level | p = 0.09 |
|  |  |
| MR-proADM ~ CRP | |
| Difference between areas | 0.16 |
| Significance level | p=0.03 |
| MR-proADM ~ Ferritin | |
| Difference between areas | 0.07 |
| Significance level | p=0.35 |
| CRP~ Ferritin | |
| Difference between areas | 0.08 |
| Significance level | p=0.17 |
| CRP~ SOFA Score | |
| Difference between areas | 0.050 |
| Significance level | p=0.39 |
| Ferritin ~ SOFA score | |
| Difference between areas | 0.03 |
| Significance level | p=0.48 |

**Supplementary Table 6.** Pairwise comparison of ROC curves of laboratory markers for ARDS development

**Supplementary Table 7.** Median laboratory values in patients in non survivors versus survivors during follow-up

| Variables | Non survivors | Survivors | p-value^*^ |
| --- | --- | --- | --- |
| MR-proADM, nmol/L (IQR) | 5.25 (2.67-6.53) | 1.15 (0.57-1.85) | p<0.0001 |
| CRP, mg/dL (IQR) | 10.52 (5.87-23.97) | 2.06 (0.53-6.11) | p<0.0001 |
| Ferritin, ng/mL (IQR) | 1463.57 (557.90- 2251.50) | 279.59 (119.30-630.40) | p=0.0037 |
| SOFA score (IQR) | 7 (4-9) | 2 (1-4) | p<0.0001 |

**^*^**Mann-Whitney Test

**Supplementary Table 8.** Accuracy of cut-off values of laboratory markers for 30-day mortality

| Variables | Cut-off | Sensitivity | Specificity | +LR | -LR | AUC | p-value |
| --- | --- | --- | --- | --- | --- | --- | --- |
| MR-proADM, nmol/L | >2 | 78.57 | 88.00 | 6.55 | 0.24 | 0.89 | <0.0001 |
| CRP, mg/dL | >2.91 | 100.00 | 57.69 | 2.36 | 0.23 | 0.85 | <0.0001 |
| Ferritin, ng/mL | >635.86 | 75.00 | 76.47 | 3.19 | 0.33 | 0.77 | 0.0016 |
| SOFA score | >3 | 81.25 | 74.51 | 3.19 | 0.25 | 0.85 | <0.0001 |

**Supplementary Table 9.** Pairwise comparison of ROC curves of laboratory markers for 30-day mortality

| MR-proADM ~ SOFA score | |
| --- | --- |
| Difference between areas | 0.0417 |
| Significance level | p = 0.5041 |
|  |  |
| MR-proADM ~ CRP | |
| Difference between areas | 0.073 |
| Significance level | p=0.34 |
| MR-proADM ~ Ferritin | |
| Difference between areas | 0.122 |
| Significance level | p=0.13 |
| CRP~ Ferritin | |
| Difference between areas | 0.049 |
| Significance level | p=0.52 |
| CRP~ SOFA Score | |
| Difference between areas | 0.036 |
| Significance level | p=0.58 |
| Ferritin ~ SOFA score | |
| Difference between areas | 0.085 |
| Significance level | p=0.31 |

**Supplementary Table 10.** Cox regression model for 30-day mortality in patients with MR-proADM values ≥ 2 nmol/L

| Variables | HR | 95% CI | p-value |
| --- | --- | --- | --- |
| MR-proADM | 12.34 | 2.66-57.28 | < 0.01 |
| Age | 1.02 | 0.98-1.08 | 0.24 |
| Male sex | 0.83 | 0.28-2.47 | 0.74 |

**Supplementary Figure 1** ROC curves for laboratory marker in patients with SARS-CoV2 infection

**
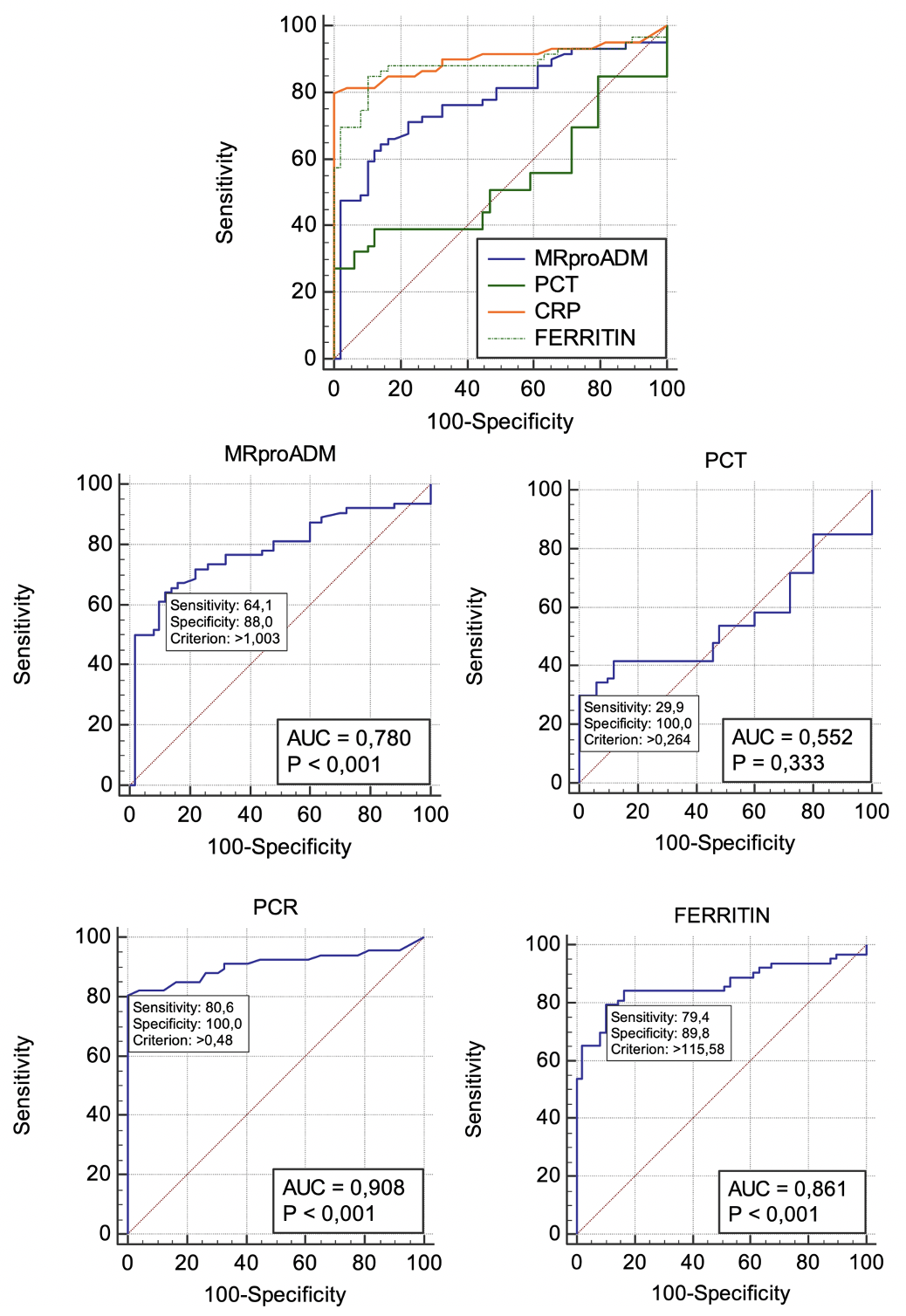
**

MR-proADM, Mid-regional proadrenomedullin; PCR, C-reactive protein; PCT, procalcitonin; ROC, receiver operating characteristic

**Supplementary Figure 2** ROC curves for ARDS development


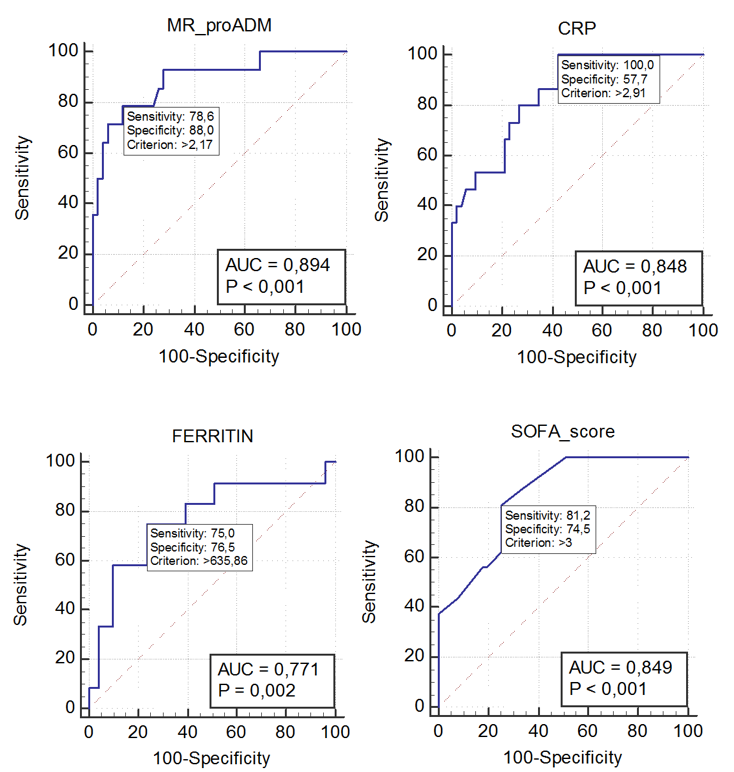


ARDS, acute respiratory distress syndrome; MR-proADM, Mid-regional proAdrenomedullin; PCR, C-reactive protein, ROC, receiver operating characteristic; SOFA, sequential organ failure assessment

**Supplementary Figure 3** ROC curves for 30-day mortality


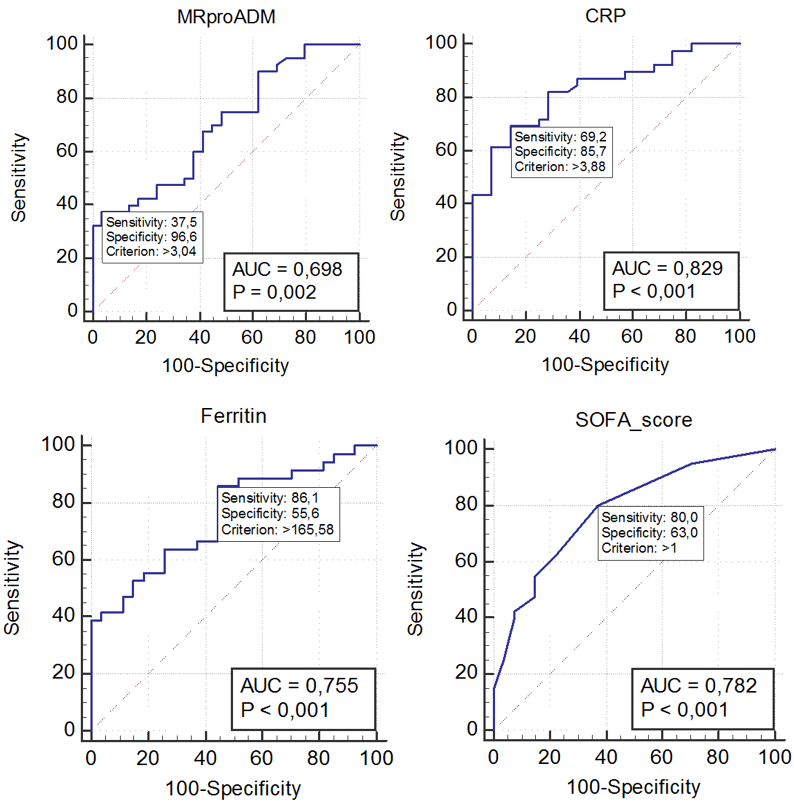


MR-proADM, Mid-regional proAdrenomedullin; PCR, C-reactive protein, ROC, receiver operating characteristic; SOFA, sequential organ failure assessment
